# Behavioral and psychological symptoms of dementia and Alzheimer’s disease progression in Down syndrome

**DOI:** 10.1186/s11689-025-09604-w

**Published:** 2025-04-11

**Authors:** Melissa R. Jenkins, Jamie C. Peven, Lauren Kubic, Benjamin L. Handen, Sharon J. Krinsky-McHale, Christy L. Hom, Alice Lee, Dana L. Tudorascu, Max McLachlan, Matthew Zammit, Davneet Minhas, Weiquan Luo, Charles Laymon, Joseph H. Lee, Ira Lott, Annie Cohen, Beau M. Ances, H. Diana Rosas, Florence Lai, Shahid H. Zaman, Elizabeth Head, Mark Mapstone, Bradley T. Christian, Sigan L. Hartley, the Alzheimer Biomarker Consortium - Down syndrome

**Affiliations:** 1 Waisman Center, University of Wisconsin-Madison, Madison, WI 53705 USA; 2 Department of Psychiatry, University of Pittsburgh, Pittsburgh, PA 15213 USA; 3 Behavioral Health Service Line, VA Pittsburgh Healthcare System, Pittsburgh, PA 15240 USA; 4 University of Pittsburgh Medical Center, University of Pittsburgh, Pittsburgh, PA 15213 USA; 5 Department of Psychology, New York State Institute for Basic Research in Developmental Disabilities, Staten Island, NY 10314 USA; 6 Department of Psychiatry & Human Behavior, Irvine School of Medicine, University of California, Irvine, CA 92697 USA; 7 Department of Medical Physics, University of Wisconsin–Madison, Madison, WI 53705 USA; 8 Department of Radiology, University of Pittsburgh, Pittsburgh, PA 15240 USA; 9 Department of Bioengineering, University of Pittsburgh, Pittsburgh, PA 15240 USA; 10 Taub Institute for Research on Alzheimer’s Disease and the Aging Brain, Sergievsky Center, and Department of Neurology, Vagelos College of Physicians and Surgeons, Columbia University, New York, NY 10032 USA; 11 Department of Neurology, Irvine School of Medicine, University of California, Irvine, CA 92617 USA; 12 Department of Neurology, Washington University in St. Louis, St. Louis, MO 63130 USA; 13 Department of Neurology, Massachusetts General Hospital, Harvard Medical School, Boston, MA 02114 USA; 14 Department of Psychiatry, University of Cambridge, Cambridge, UK; 15 Department of Pathology & Laboratory Medicine, Irvine School of Medicine, University of California, Irvine, CA 92617 USA; 16 Department of Neurology, Irvine School of Medicine, University of California, Irvine, CA 92697 USA; 17 Department of Psychiatry, University of Wisconsin-Madison, Madison, WI 53705 USA; 18 School of Human Ecology, University of Wisconsin-Madison, Madison, WI 53705 USA

**Keywords:** Down syndrome, Alzheimer’s disease, Amyloid, Tau, Psychiatric symptoms

## Abstract

**Background:**

Adults with Down syndrome (DS) have a 90% lifetime risk for Alzheimer’s disease (AD), with neurobiological pathology present decades prior to dementia onset. The profile and timing of cognitive decline in DS is well-documented. However, there is a small body of research on whether Behavioral and Psychological Symptoms of Dementia (BPSD) occur early on in the progression of AD in DS and are associated with early AD pathology (i.e., amyloid-beta [Aβ] and neurofibrillary tau tangles [NFT]).

**Methods:**

Data were analyzed from 337 adults with DS (*M* = 45.13 years, *SD* = 9.53 years) enrolled in a large cohort study. The Reiss Screen for Maladaptive Behavior (RSMB) measured common behaviors reported in BPSD across up to four study cycles (spaced approximately 16 months apart). Linear mixed models estimated change in BPSD as predicted by baseline (a) dementia status (i.e., cognitively stable, mild cognitive impairment [MCI], or dementia), (b) Aβ positron emission tomography (PET) tracer [^11^C] PiB, and (c) NFT PET tracer [^18^F]AV-1451. Models controlled for chronological age, sex, study site, premorbid intellectual disability level, APOE e4 allele carrier status, psychiatric diagnoses, and psychiatric medication use.

**Results:**

Compared to cognitively stable participants, participants whose status was MCI or dementia, had significantly higher baseline RSMB subdomain scores. Increases in RSMB Depression-Behavioral, Depression-Physical, and Psychosis were observed for participants with MCI. Higher baseline Aβ and NFT were associated with higher RSMB Avoidant at baseline, and increases in RSMB Depression-Physical and Psychosis over time.

**Conclusions:**

BPSD are an important part of AD in DS, particularly during the prodromal stage. Elevated Aβ and NFT predict higher initial avoidance and change in physical depression behaviors and may indicate MCI in adults with DS. Broader increases in BPSD are observed as adults with DS progress from early to late-stage dementia. Clinicians should rule out other possible causes of BPSD when screening for AD, such as stressful life experiences or co-occurring medical conditions. Caregivers of adults with DS should have resources on BPSD management and self-care strategies.

**Supplementary Information:**

The online version contains supplementary material available at 10.1186/s11689-025-09604-w.

Individuals with Down syndrome (DS) have a high risk for Alzheimer’s disease (AD) due to the triplication of chromosome 21, which carries the amyloid precursor protein gene (APP) [1–4]. This results in the overexpression of beta-amyloid (Aβ) that accumulates into extracellular plaques in the brain, which is a hallmark characteristic of AD [1–3]. The development of Aβ plaques in the DS population typically begins in the mid to late 30s [1, 2], and in some cases earlier [5], which is much younger than in sporadic late onset AD (LOAD) [6] and similar to autosomal dominant AD (ADAD) [7]. Consistent with the National Institute on Aging and Alzheimer's Association (NIA-AA) research framework [8], the accumulation of Aβ plaques followed by neurofibrillary tau tangles (NFT) and then biomarkers of neurodegeneration in DS [9]. On average, individuals with DS evidence AD dementia in their mid-50s [10, 11] with impairments in memory, attention, and visuospatial skills among the earliest affected cognitive domains [12–14], and the onset of clinical dementia occurring following NFT deposition and neurodegeneration [15, 16].

In both LOAD and ADAD, AD clinical expression includes Behavioral and Psychological Symptoms of Dementia (BPSD) such as sadness, anxiousness, paranoia, and agitation [17–19]. Often these symptoms emerge after a dementia diagnosis [20–22]. However, other studies report that depressive symptoms including apathy occur prior to onset of dementia, during what has been described as part of mild behavioral impairment (MBI) [23, 24]. The MBI is an analogue to mild cognitive impairment (MCI), which describes early and subtle cognitive declines that precede dementia [25]. Indeed, in general population studies, increases in the frequency of some BPSD (e.g., agitation, social withdrawal, and sadness or apathetic mood) begin years before an AD dementia diagnosis [26, 27] and are associated with elevated Aβ plaques and NFT [28, 29]. Other BPSD changes are reported at later stages of AD progression including anxiety, aggression, avoidant behaviors, and disruptive behaviors [30, 31].

Across the life course, people with DS exhibit more behavioral and psychological symptoms than their neurotypical peers in the general population [32–35]. Increases in or new behavioral problems and psychological symptoms, indicative of BPSD, have been reported to be part of the early stages of AD in DS [36–38]. Indeed, in particular, BPSD involving anxious behavior, apathy, social withdrawal or other depressive symptoms (e.g., low or sad mood, appetite disturbance, psychomotor slowing) has been reported by caregivers—as being among the first signs of possible dementia in DS [37, 39], potentially occurring before memory loss [36, 40]. In other studies with people with DS, apathy and other depressive symptoms were reported at the time of AD dementia onset. In a sample of 251 adults with DS, Urv et al. [41] reported that adults with DS who converted to AD dementia (DSAD) display increases in depressive symptoms when compared with those without dementia. High levels of agitation, restlessness, and hyperactivity coincide with early-stage dementia in other cohorts of adults with DS [42–44]. Increases in aggression are reported to be part of AD dementia in some [41, 43], but not other DS studies [45, 46].

Outside of DS, it has been posited that Aβ plaques and NFT are neurobiological mechanisms that drive BPSD including apathy and depression symptoms [28, 47]; thus, BPSD are hypothesized to be driven by early AD disease progression, with onset before dementia onset. To date little is known about the relation between Aβ plaques, NFT, and BPSD in DS. Prior studies [41, 43, 44, 48] have been largely cross-sectional, focused on later-stage disease progression (i.e., after onset of dementia) and did not include biomarkers of AD pathology. A better understanding of BPSD across AD disease progression in DS is needed to inform screening practices (i.e., which BPSD should be screened for and when) and guide the development of treatment options and care planning (e.g., how to manage symptoms that interfere with daily functioning).

The goal of the current study was to describe changes in BPSD as AD unfolds in aging individuals with DS by leveraging up to four cycles of data collection spanning a total of up to 5.5 years from the Alzheimer Biomarker Consortium-Down Syndrome (ABC-DS). The research objectives were to: 1) determine differences in BPSD based on clinical AD status (i.e., cognitively stable, MCI, and dementia); and 2) examine the association between neuroimaging biomarkers of early AD pathology (i.e., Aβ PET and tau PET) and BPSD in individuals with DS. For the first research objective, it was hypothesized that adults with DS who were cognitively stable at baseline would exhibit less initial BPSD and less increase in BPSD over time compared to those with MCI and dementia. For the second research objective, it was hypothesized that greater baseline Aβ and tau would be associated with more initial BPSD and greater increase in BPSD over time. Based on findings in LOAD [49, 50], it was predicted that increases in depressive symptoms would occur early in AD progression, and be related to higher Aβ and tau in adults with DS.

## Methods

### Participants

Analyses included 337 adults with DS who were enrolled in the ABC-DS [51], an ongoing multisite (nine data collection sites in the U.S. and one site in the U.K.) longitudinal study aimed at characterizing imaging, biofluid, behavioral, and cognitive changes associated with the DSAD. Within the ABC-DS study, eligible participants were at least 25 years old, had a mental age of at least three years (determined by standardized IQ tests or medical records), had a study partner who was able to report on their medical history and functioning, and had their DS status and karyotyping confirmed through genetic testing. All participants or their legal guardian provided consent in accordance with the Declaration of Helsinki. If consent could not be provided, assent was obtained.

### Procedures

Participants completed up to four time points of data collection, with study visits occurring between 2015 and 2024. Each data collection cycle was spaced approximately 16 months apart (16 ± 3 months) based on study design. At each data collection cycle, the participant with DS was administered a comprehensive neuropsychological battery and underwent magnetic resonance imaging (MRI) and positron emission tomography (PET) to measure AD neuropathological burden (i.e., Aβ and tau) as well as blood draws to measure biofluid biomarkers and for genetics analyses. The MRI was conducted to interpret PET imaging scans. Study partners reported on the participants’ socio-demographics, medical history, recent life events, and completed questionnaires about BPSD and functioning. Each participant’s clinical AD status was determined based on a case consensus approach described below.

## Measures

### Socio-demographic characteristics

Participants’ socio-demographic information was collected at the baseline visit and included age in years, biological sex (coded: female = 0, male = 1), race (Non-White = 0, White = 1), and ethnicity (Non-Hispanic = 0, Hispanic = 1). Apolipoprotein E (APOE) carrier status (ɛ4 allele absent = 0, ɛ4 allele present = 1) was determined through genetic testing. Any psychiatric diagnoses (coded: no = 0, yes = 1) and any psychiatric medication use (coded: no = 0, yes = 1) at each data collection cycle was also recorded. Premorbid intellectual disability (ID) level was based on IQ and adaptive behavior testing results reported in medical records or through the Stanford-Binet Intelligence Scales, Fifth Edition (SB5) [52] Abbreviated Battery and Vineland Adaptive Behavior Scales, Third Edition (VABS) [53]. Mild ID (coded 0) was defined as IQ scores between 50–69 and commensurate VABS score, moderate ID (coded 1) as IQ between 35–49 and commensurate VABS score, and severe/profound ID (coded 2) as IQ < 35 and commensurate VABS score. The lowest possible SB5 Abbreviated IQ score is 40 and thus mental age 3 was used to differentiate individuals with moderate from severe/profound ID.

### Clinical AD status

Clinical AD status was determined via a consensus approach that included a psychologist, physician, and at least two other members of the research team with expertise in AD in DS. Individuals involved in these consensus conversations were blind to neuroimaging and biofluid AD biomarker data. Clinical AD status was based on review of all available study partner-reported measures of daily functioning, medical history, and directly-administered neuropsychological measures. Decisions considered premorbid ID and recent stressful live events. Participants were classified as either: (1) cognitively stable, (2) MCI, (3) dementia, or (4) unable to determine [51]. Cognitively stable participants did not show signs of dementia-related decline. Participants with MCI evidenced mild decline in cognitive skills on some but not all directly-administered cognitive measures but did not evidence significant declines in their functional ability as reported by their study partner. Participants with dementia evidenced marked cognitive declines across directly administered measures and were reported by caregivers to have marked functional declines. A status of unable to determine was given if cognitive level was unclear and/or if cognitive or functional decline were present but medical conditions or recent life events could not be ruled out as the cause. For these reasons, this status group was excluded from analyses. Full details on the clinical consensus process have been published [51].

### BPSD symptoms—Reiss Screen for Maladaptive Behavior

The Reiss Screen for Maladaptive Behavior (RSMB) is a 38-item questionnaire that measures internalizing and externalizing behavior problems in individuals with ID [54]. Study partners are asked to rate each item based on the participants’ behavior in the past 2 to 3 months as: “no problem” (i.e., does not apply or is not sufficiently frequent, intense, or severe; scored 0), a “problem” (i.e., causes significant discomfort and interference in daily functioning; scored 1), or a “major problem” (i.e., causes great suffering and/or occurs with very high frequency and intensity; scored 2). The RSMB includes eight subdomains: (a) Aggression (e.g. physical or verbal attacks on others), (b) Autism (e.g., repetitive movements that are non-functional) which we renamed Restricted and Repetitive Behaviors, (c) Psychosis (e.g., beliefs that are not based on reality), (d) Paranoia (e.g., mistrust and suspicion of others), (e) Depression—Behavior Symptoms (e.g., crying spells), (f) Depression- Physical Symptoms (e.g., lacks energy), (g) Dependent Personality (e.g., excessive reliance on others), and (h) Avoidant (e.g., avoids interactions with others). These subdomains are not intended to screen for clinically significant psychiatric conditions but rather assess for the presence and severity of symptoms. Subdomain scores and RSMB Total score were used in analyses to reflect level of BPSD (i.e. frequency and severity). Prior studies provide evidence of good concurrent validity of the RSMB with other instruments measuring challenging behaviors, including the Adaptive Behavior Scale Part II and Aberrant Behavior Checklist [55, 56]. However, it should be noted that the RSMB was not developed to screen for BPSD.

### MRI processing

MRI scans were used to process and interpret PET scans (described below) and were acquired on a 3 T GE Discovery MR750, Siemens Trio, Siemens Prisma, or GE Signa PET/MR depending on the imaging site. High-resolution T1-weighted (T1W) images were acquired using a 3D fast spoiled gradient echo (FSPGR) or magnetization prepared rapid acquisition gradient echo (MPRAGE) sequence. MRIs were acquired at the same visit as the individual’s neuropsychological evaluation. Previous work has described the processing techniques for MRI scans in this cohort [51]. In brief detail, a subset of T1w scans was parcellated using FreeSurfer v5.3.0, producing native space versions of the Desikan-Killiany atlas for each scan [57]. Results were inspected, and 12 high-quality parcellations were selected as templates. The 12 templates were warped into each participant’s native MR space using the Advanced Neuroimaging Tools (ANTs) software package [58, 59]. A final native space atlas was created for each scan by determining the maximum overlap of each parceled region from the 12 warped templates. All results were accepted or rejected based on a visual rating of the final atlas’ adherence to the MR anatomy of the participant. In a few cases, if the multi-template method did not produce acceptable parcellations, the direct application of FreeSurfer on the scan was used. Each participant-specific atlas was used to construct the six Braak regions [60, 61].

### PET processing

PET scans were acquired on a variety of PET and PET/CT platforms with each certified for multi-center studies, as described in Handen et al. [51] The amyloid PET scans were acquired with either [11C]PiB or [18F]florbetapir measuring PET Aβ signal 50–70 min post-injection. The tau PET scans were acquired with [18F]AV-1451, measuring PET NFT signal 80–100 min post-injection. PET images were acquired in 5-min frames, corrected for motion on a frame-by-frame basis using SPM8, and time averaged. The amyloid PET scans were used to calculate global amyloid burden with the Centiloid method [62] using SPM8 software. PET scans were harmonized across study sites [51] and PET images were registered with their corresponding anatomical MR images. The MR scan then underwent deformable registration to the 152-subject Montreal Neurological Institute (MN152) template. Co-registered PET images were warped into the MNI152 template space using the resulting transformation matrix. Standard uptake value ratios (SUVR) were calculated for the standard global region, using the whole cerebellum for reference, and converted to Centiloids (CL) with the published Eq [62]. The [^18^F]AV-1451 NFT images were used to calculate NFT burden; PET images were similarly registered with corresponding structural T1 MRIs. Concentration of [^18^F]AV-1451 was expressed as SUVR in the parcellation-defined Braak regions [60], using cerebellar grey matter as reference. Braak stage II NFT burden has shown modest sensitivity to neuropathological changes [63]. Thus, for the current analyses, SUVR in Braak stages I (transentorhinal cortex) and II (entorhinal cortex and hippocampus) were used as a combined indicator of early NFT burden.

### Data analysis

Statistical analyses were completed using SAS version 9.4 (SAS Institute Inc., Cary, NC) and data visualization was completed using SPSS version 29 (IBM SPSS Statistics). Descriptive statistics were used to characterize the study sample. Linear mixed effects models using restricted maximum likelihood (REML) were estimated to explore longitudinal associations while controlling for and examining between-person differences in BPSD progression. Specifically, we tested within-person changes in RSMB scores predicted by (a) clinical AD status, and (b) AD biomarkers of PET Aβ and PET NFT. SAS PROC MIXED was used for modeling fixed effects with random intercept terms and a random effect for study site location. Time (latency in years since baseline) was entered in level 1. Chronological age (in years) mean-centered at baseline, sex, premorbid ID level, APOE e4 allele carrier status, psychiatric diagnoses, and psychiatric medication use were entered as covariates at level 2. Maximum likelihood ratio (−2ΔLL) tests were conducted to see if adding fixed interaction effects between time and the three predictors of interest (i.e., clinical AD status, PET Aβ, and PET NFT) improved model fit. Quadratic models were tested to examine potential non-linear associations between the main predictors and outcomes. The model fit did not improve when including quadratic functions. See Supplemental Statistical Models for each equation. Separate moderation analyses were also conducted to test the impact of fixed interaction effects between time and four covariates (i.e., age, sex, premorbid ID, and APOE e4 allele carrier status) on model fit in analyses of RSMB scores predicted by clinical AD status.

## Results

Table 1 includes socio-demographic information for all study participants according to clinical AD status. Supplemental Table 1 provides the mean and SD for RSMB scores by clinical AD status across all data collection cycles. Of the 337 participants, 222 (65.9%) underwent Aβ PET scans and 143 (42.4%) also underwent tau PET scans. One individual was excluded from the clinical AD status and Aβ analyses due to missing covariate information. At baseline, 73.3% (*n* = 247) were cognitively stable, 14.2% (*n* = 48) had MCI, and 12.5% (*n* = 42) had dementia. On average, participants were 45 years old (*M* = 45.13, *SD* = 9.53), 54.3% were male, 96.7% were White, and 95.8% were non-Hispanic. Premorbid ID levels were: 47.2% mild, 44.2% moderate, and 8.6% severe or profound. Approximately one-fourth of participants (24.9%) were APOE e4 allele carriers. At baseline average RSMB Total score was 3.15 (SD = 4.60); the highest average RSMB subdomain score was for Depression-Physical (*M* = 0.93, *SD* = 1.48), while the lowest average RSMB subdomain score was for Restricted and Repetitive Behaviors (*M* = 0.32, *SD* = 0.75). Participants without Aβ PET or tau PET data did not significantly differ from participants with imaging data with respect to APOE e4 allele carrier status or premorbid ID levels. However, participants with Aβ PET scans were younger (*M* = 42.63, *SD* = 9.46) than those without Aβ PET scans (*M* = 49.95, *SD* = 7.65), t(276.83) = −7.66, *p* < 0.001. Similarly, participants with tau PET were younger (*M* = 38.73, *SD* = 8.34) than those without tau PET (*M* = 49.84, *SD* = 7.37), t(335) = −12.93, *p* < 0.001. More male participants had Aβ PET scans than female participants, χ^2^ (1, *N* = 337) = 6.97, *p* = 0.008.

**Table 1:** Baseline sample demographics across clinical AD status

	Cognitively stable		MCI		Dementia	
Variables	*N* or *M*	% or *SD*	*N* or *M*	% or *SD*	*N* or *M*	% or *SD*
Sex						
Male	132	53.44	33	68.75	18	42.86
Race						
White	237	97.13	45	93.75	40	97.56
Ethnicity						
Non-Hispanic	234	94.74	48	100	41	97.62
Intellectual Disability						
Mild	121	48.99	20	41.67	18	42.86
Moderate	106	42.91	24	50.00	19	39.58
Severe	20	8.10	4	8.33	5	11.90
APOE4						
Yes	49	19.92	17	35.42	18	42.86
Age (years)	42.20	9.00	52.17	5.60	54.31	5.08
RSMB						
Aggression	0.43	1.11	0.69	1.43	1.33	2.18
Restricted/Repetitive	0.25	0.69	0.56	0.92	0.48	0.77
Depression-Behavior	0.46	1.08	0.71	1.41	1.02	1.09
Depression-Physical	0.74	1.32	1.15	1.35	1.76	2.09
Avoidant Personality	0.41	0.82	0.69	1.22	0.98	1.26
Dependent Personality	0.59	1.15	0.85	1.40	1.05	1.29
Psychosis	0.32	0.83	0.54	0.90	1.02	1.07
Paranoia	0.32	0.79	0.58	1.24	0.62	1.19
Total Score	2.47	4.03	3.90	4.80	6.31	5.97

*RSMB* Reiss Screen for Maladaptive Behavior, *AD* Alzheimer’s Disease, *MCI* Mild cognitive impairment

### Clinical dementia status predicting BPSD

**Fig. 1:**
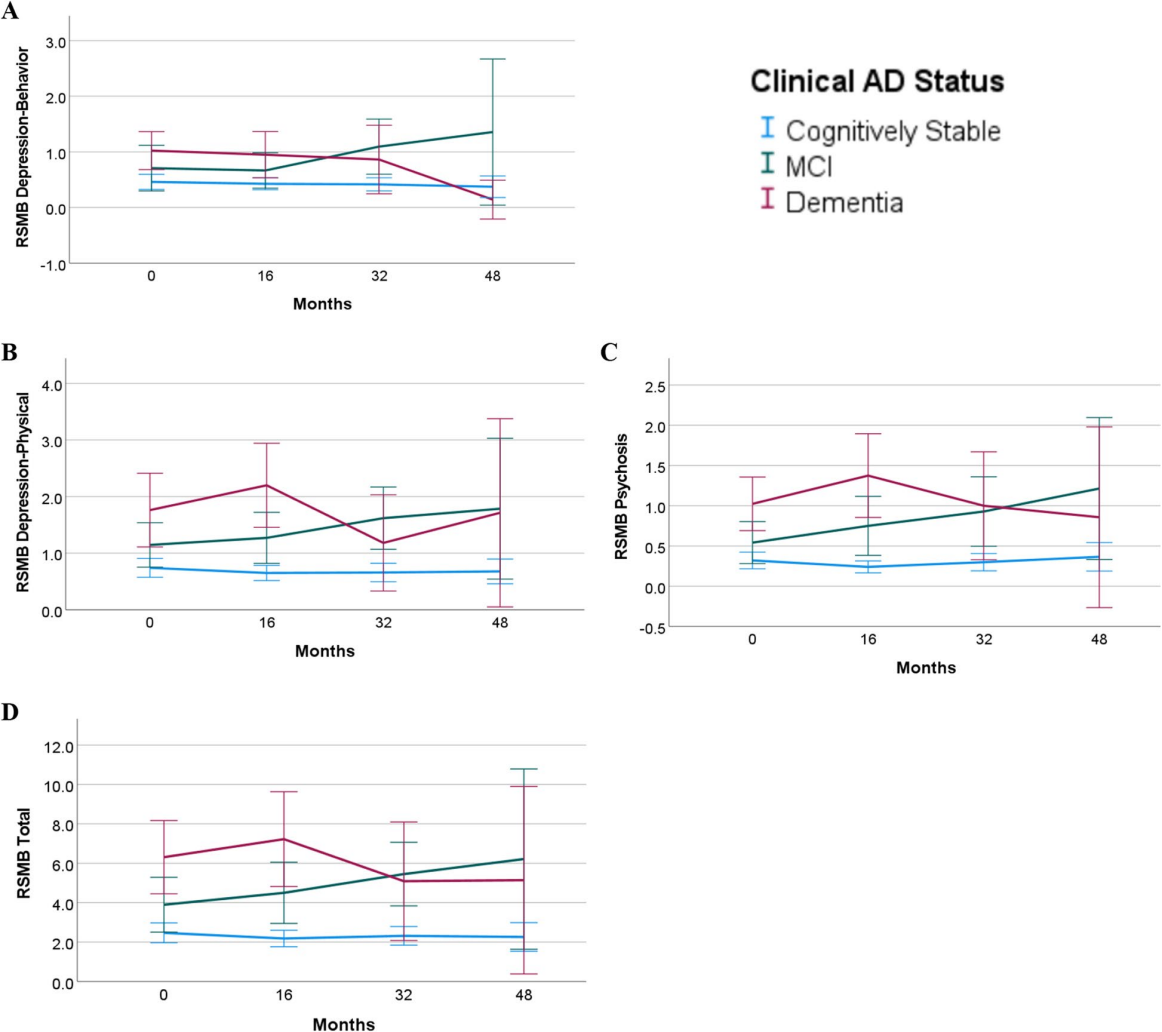
Marginal Means of RSMB Scores by Baseline Clinical AD Status. Note*.* Marginal means for the Reiss Screen of Maladaptive Behavior (RSMB) across four data collection cycles for depression-behavior (**A**) depression-physical (**B**), psychosis (**C**), and total (**D**) for cognitively stable (blue), mild cognitive impairment (green), and dementia (red)

Mixed effects models examining differences in RSMB scores predicted by clinical AD status are presented in Table 2. The log-likelihood ratio tests were significant (*p* < 0.05) for all RSMB subdomains except for Aggression, Restricted and Repetitive Behaviors, Avoidant, Dependent Personality, and Paranoia; thus, interactions between baseline clinical AD status and study cycle to predict BPSD change were examined for Depression-Behavior, Depression-Physical, Psychosis, and Total RSMB. Main effects for clinical AD status were statistically significant for participants with dementia (Aggression, Avoidant, Dependent Personality, and Paranoia) and MCI (Restricted and Repetitive Behaviors, Avoidant, and Paranoia), such they had higher levels of these BPSD than cognitively stable participants. In models with interaction terms, participants with MCI at baseline compared to those who were cognitively stable had significantly greater changes in Depression-Behavior (*b* = 0.175, *p* < 0.001, 95% CI [0.077, 0.273]), Depression-Physical (*b* = 0.164, *p* < 0.01, 95% CI [0.042, 0.286]), Psychosis (*b* = 0.120, *p* < 0.01, 95% CI [0.034, 0.205]), and Total RSMB (*b* = 0.504, *p* < 0.01, 95% CI [0.146, 0.862]). Figure 1 depicts the relation between baseline clinical AD status and average change in RSMB subdomains and Total RSMB across time, unadjusted for covariates. Relative to participants who were cognitively stable at baseline, participants who had a clinical AD status of dementia did not have any significantly different changes in BPSD. Males had significantly lower Depression-Behavior (*b* = −0.309, *p* < 0.001, 95% CI [−0.464, −0.154]), Dependent Personality (*b* = −0.313, *p* < 0.001, 95% CI [−0.494, −0.131]), and Total RSMB (*b* = −0.862, *p* = 0.02, 95% CI [−1.570, −0.153]) scores than females. Psychiatric medication use was statistically significant in all models except for Depression-Behavior, and psychiatric diagnoses was significant in all models except for Restricted and Repetitive Behaviors, Avoidant, and Psychosis. Additionally, model fit improved for two moderation analyses with fixed interaction effects between time and the covariates premorbid ID and APOE4. Individuals with severe ID experienced a greater change in Depression-Behavior over time than individuals with mild ID (*b* = 0.148, *p* = 0.02, 95% CI [0.035, 0.261]), and APOE4 allele carriers experienced lower Aggression scores over time than non-carriers (*b* = −0.07, *p* = 0.05, 95% CI [−0.138, −0.001]).

**Table 2:** Multilevel models of clinical AD status at baseline predicting RSMB

		Estimates				
RSMB	Fixed Effects	*B*	*SE*	*t*	*df*	95% CI
Aggression	Intercept	0.176	0.113	1.56	328	[−0.046, 0.398]
	Time	0.001	0.017	0.350	728	[−0.028, 0.040]
	MCI	0.279	0.168	1.660	728	[−0.051, 0.608]
	Dementia	0.591	0.187	3.160**	728	[0.223, 0.958]
Restricted/Repetitive	Intercept	0.060	0.066	0.910	328	[−0.070, 0.190]
	Time	0.015	0.012	1.260	729	[−0.008, 0.037]
	MCI	0.315	0.095	3.321**	729	[0.128, 0.501]
	Dementia	0.138	0.107	1.280	729	[−0.073, 0.349]
Depression-Behavior	Intercept	0.551	0.093	5.920***	328	[0.368, 0.734]
	Time x MCI	0.175	0.050	3.510***	725	[0.077, 0.273]
	Time x Dementia	−0.097	0.059	−1.63	725	[−0.213, 0.020]
Depression-Physical	Intercept	0.541	0.122	4.420***	328	[0.300, 0.781]
	Time x MCI	0.164	0.062	2.630**	727	[0.042, 0.286]
	Time x Dementia	−0.056	0.075	−0.740	727	[−0.202, 0.091]
Avoidant	Intercept	0.215	0.098	2.190*	328	[0.022, 0.407]
	Time	0.037	0.016	2.230*	729	[0.004, 0.069]
	MCI	0.557	0.141	3.940***	729	[0.279, 0.835]
	Dementia	0.579	0.160	3.630***	729	[0.266, 0.892]
Dependent	Intercept	0.526	0.099	5.330***	328	[0.332, 0.720]
	Time	−0.010	0.016	−0.610	729	[−0.042, 0.022]
	MCI	0.281	0.143	1.960	729	[−0.001, 0.562]
	Dementia	0.384	0.161	2.380*	729	[0.068, 0.701]
Psychosis	Intercept	0.255	0.082	3.130**	328	[0.094, 0.415]
	Time x MCI	0.120	0.043	2.750**	727	[0.034, 0.205]
	Time x Dementia	0.005	0.052	0.100	727	[−0.097, 0.107]
Paranoia	Intercept	0.233	0.078	2.959**	328	[0.080, 0.386]
	Time	0.011	0.013	0.900	729	[−0.013, 0.036]
	MCI	0.257	0.113	2.260*	729	[0.034, 0.478]
	Dementia	0.261	0.127	2.050*	729	[0.010, 0.511]
Total	Intercept	1.847	0.386	4.780***	328	[1.088, 2.607]
	Time x MCI	0.504	0.182	2.770**	727	[0.146, 0.862]
	Time x Dementia	−0.201	0.220	−0.910	727	[−0.633, 0.231]

*N* = 336. Unstandardized coefficients (*B*s) are presented. All models include a random intercept and control for study site, age at baseline, sex, APOE ϵ4 carrier status, premorbid intellectual disability, psychiatric medications, and psychiatric diagnoses. Main effects were entered into models with interactions. Cognitively stable is reference group

*RSMB* Reiss Screen for Maladaptive Behavior, *MCI* Mild cognitive impairment

^***^ *p* < .001. ** *p* < .01. * *p* < .05

Supplemental Table 2 provides cross-tabulations of participants’ change in clinical AD status over the course of the study.

### Aβ burden predicting BPSD

**Fig. 2:**
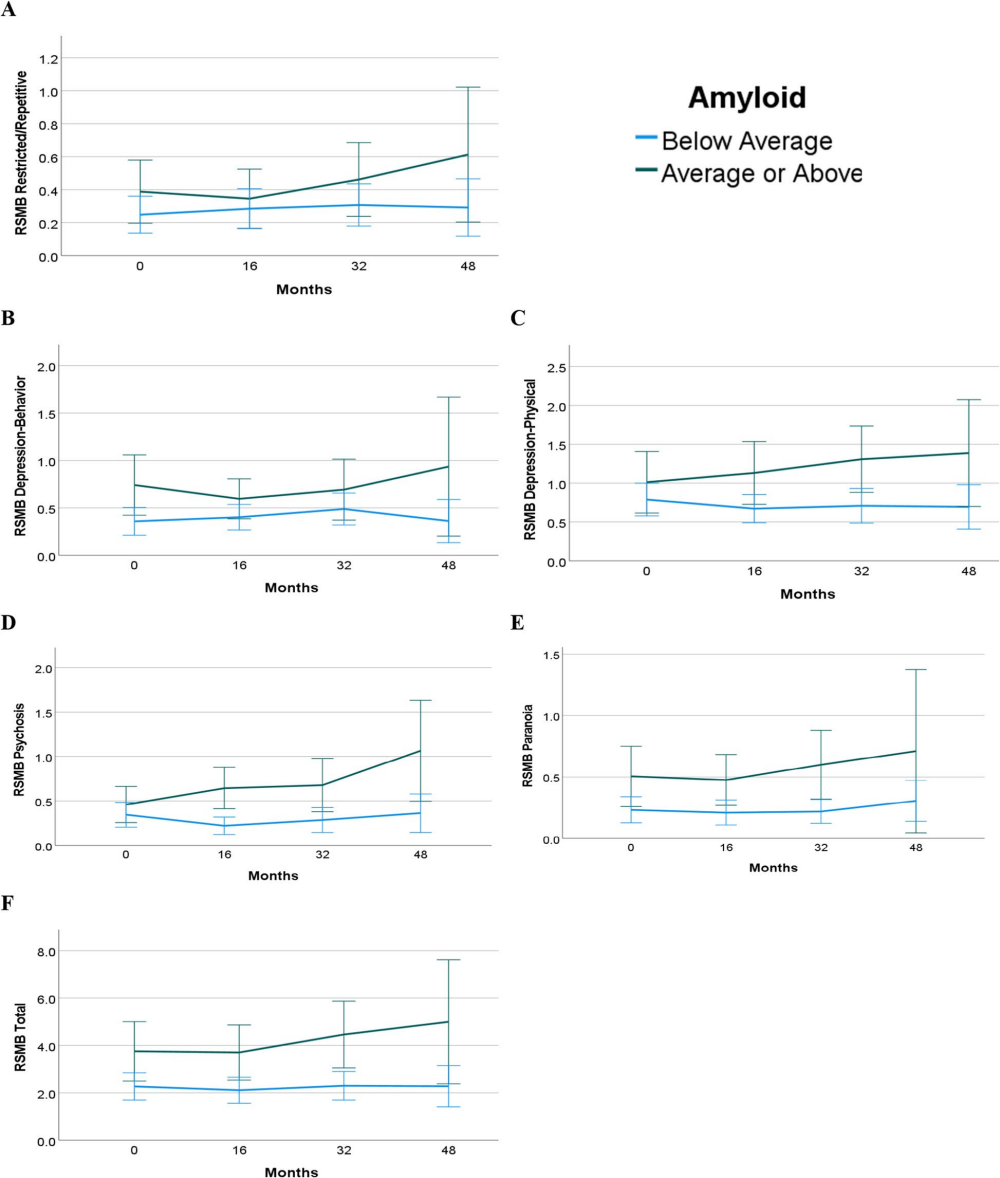
Marginal Means of RSMB Scores by Average Baseline Amyloid. Note*.* Marginal means for the Reiss Screen of Maladaptive Behavior (RSMB) across four data collection cycles for restricted and repetitive (**A**), depression-behavior (**B**), depression-physical (**C**), psychosis (**D**), paranoia (**E**), and total (**F**) for average or above average Aβ (green) and below average Aβ (blue)

Table 3 presents results of the mixed effects models with Aβ at baseline predicting RSMB scores over time in 221 participants, after controlling for relevant covariates (age, biological sex, premorbid ID, APOE allele 4 carrier status, study site, psychiatric medications and diagnosis). The log-likelihood ratio tests were significant (*p* < 0.05) for all RSMB subdomains except for Aggression, Avoidant, and Dependent Personality; thus, interactions between baseline clinical AD status and study cycle to predict BPSD change were examined for Restricted and Repetitive Behaviors, Depression-Behavior, Depression-Physical, Psychosis, Paranoia, and Total RSMB. The main effect for Aβ was statistically significant for Avoidant (*b* = 0.005, *p* = 0.04, 95% CI [0.000, 0.009]), but not for the Aggression and Dependent Personality models. For models with interactions, higher levels of Aβ at baseline were significantly associated with increase in BPSD over time for all RSMB subdomains and Total RSMB (*b* = 0.008, *p* < 0.001, 95% CI [0.004, 0.012]). Figure 2 depicts the relation between baseline Aβ and average change in RSMB subdomains and Total RSMB across time, unadjusted for covariates. For visualization, participants are grouped by low versus high Aβ using centiloid threshold of 31.17 (i.e., average or above average/below average). Males had significantly lower Depression-Behavior (*b* = −0.300, *p* < 0.01, 95% CI [−0.497, −0.104]) and Dependent Personality (*b* = −0.384, *p* < 0.01, 95% CI [−0.619, −0.149]) scores than females. Those who were APOE ϵ4 allele carriers had lower Aggression (*b* = −0.308, *p* = 0.03, 95% CI [−0.594, −0.022]) scores than non-carriers. Younger participants had higher Restricted and Repetitive Behaviors (*b* = −0.012, *p* = 0.04, 95% CI [−0.024, −0.001]) scores than older participants. Psychiatric medication use was statistically significant in all models except for Aggression, Depression Behavior, Dependent Personality, Psychosis, and Paranoia, and psychiatric diagnoses was significant in all models except for Restricted and Repetitive Behaviors.

**Table 3:** Multilevel models of amyloid at baseline predicting RSMB

	Estimates					
RSMB	Fixed Effects	*B*	*SE*	*t*	*df*	95% CI
Aggression	Intercept	0.174	0.142	1.220	214	[−0.106, 0.453]
	Time	0.009	0.016	0.590	507	[−0.022, 0.041]
	Amyloid-β	0.004	0.002	1.550	507	[−0.001, 0.008]
Restricted/Repetitive	Intercept	0.004	0.092	0.050	214	[−0.177, 0.186]
	Time x Amyloid-β	0.002	0.001	2.900**	506	[0.001, 0.003]
Depression-Behavior	Intercept	0.478	0.121	3.950***	214	[0.239, 0.716]
	Time x Amyloid-β	0.002	0.001	2.880**	506	[0.001, 0.003]
Depression-Physical	Intercept	0.599	0.177	3.390***	214	[0.250, 0.947]
	Time x Amyloid-β	0.002	0.001	2.940**	506	[0.001, 0.003]
Avoidant	Intercept	0.134	0.137	0.980	214	[−0.135, 0.403]
	Time	0.047	0.019	2.500*	507	[0.010, 0.083]
	Amyloid-β	0.005	0.002	2.010*	507	[0.000, 0.009]
Dependent	Intercept	0.521	0.137	3.800***	214	[0.251, 0.790]
	Time	0.001	0.019	−0.040	507	[−0.038, 0.036]
	Amyloid-β	0.004	0.002	1.660	507	[−0.001, 0.008]
Psychosis	Intercept	0.205	0.109	1.890	214	[−0.009, 0.419]
	Time x Amyloid-β	0.002	0.000	4.630***	506	[0.001, 0.003]
Paranoia	Intercept	0.238	0.099	2.410*	214	[0.043, 0.433]
	Time x Amyloid-β	0.002	0.000	3.470***	506	[0.001, 0.002]
Total	Intercept	1.889	0.540	3.500***	214	[0.826, 2.953]
	Time x Amyloid-β	0.008	0.002	4.000***	506	[0.004, 0.012]

*N* = 221. Unstandardized coefficients (*B*s) are presented. RSMB = Reiss Screen for Maladaptive Behavior. All models include a random intercept and control for study site, age at baseline, sex, APOE ϵ4 carrier status, premorbid intellectual disability, psychiatric medications, and psychiatric diagnoses. Main effects were entered into models with interactions

^***^ *p* < .001. ** *p* < .01. * *p* < .05

### Tau burden predicting BPSD

**Fig. 3:**
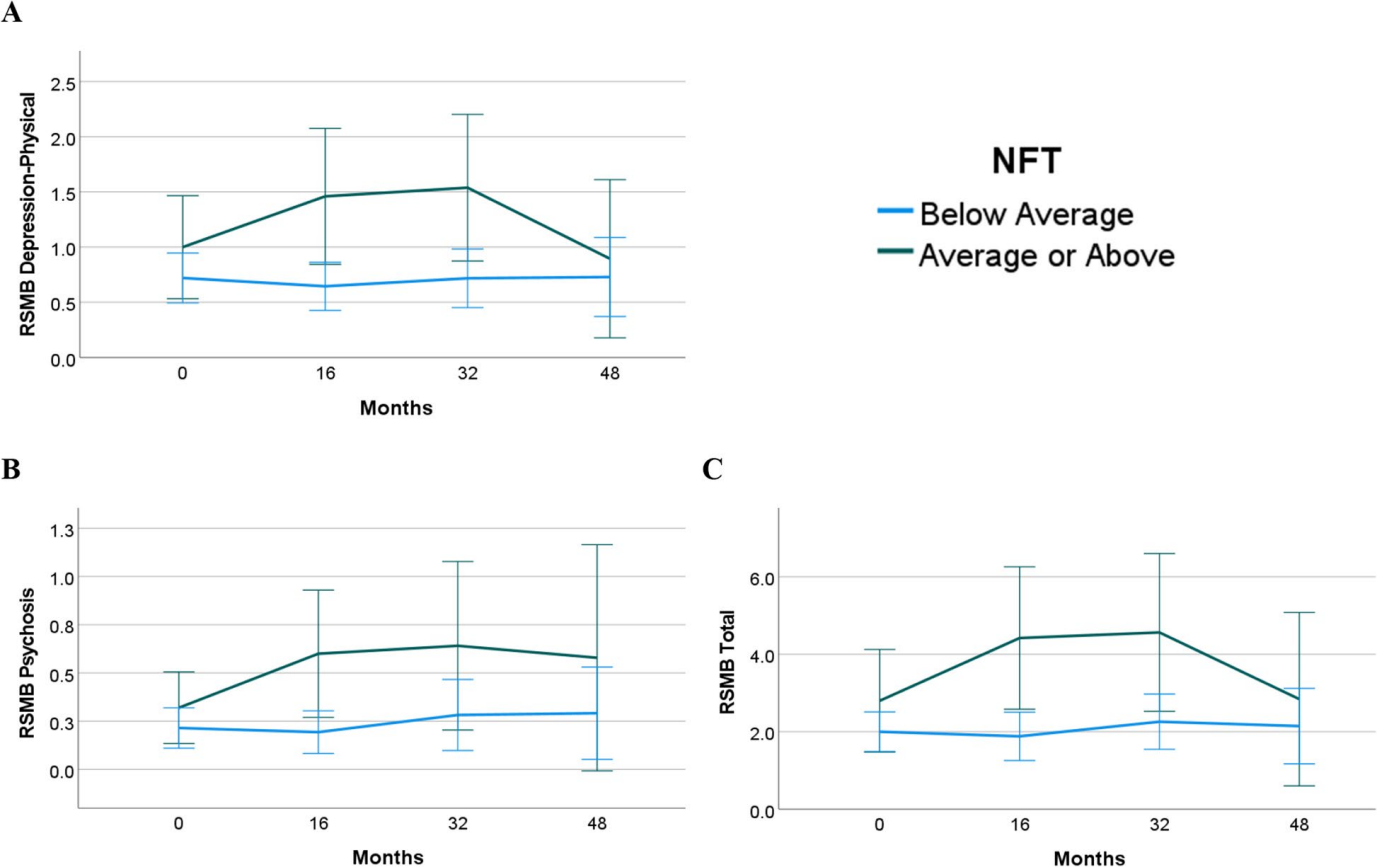
Marginal Means of RSMB Scores by Average Baseline Tau. Note*.* Marginal means for the Reiss Screen of Maladaptive Behavior (RSMB) across four data collection cycles for depression-physical (**A**), psychosis (**B**), and total (**C**) for average or above average neurofibrillary tau tangles (NFT) (green) and below average NFT (blue)

Table 4 presents results of the mixed effects models with tau at baseline predicting RSMB scores over time in 143 participants, after controlling for relevant covariates. The log-likelihood ratio tests were significant (*p* < 0.05) for all RSMB subdomains except for Aggression, Restricted and Repetitive Behaviors, Depression-Behavior, Avoidant, Dependent Personality, and Paranoia; thus, interactions between tau and study cycle to predict BPSD change were examined for Depression-Physical, Psychosis, and Total RSMB. Main effects for tau were statistically significant for Restricted and Repetitive Behaviors (*b* = 0.540, *p* = 0.05, 95% CI [0.006, 1.073]), Depression-Behavior (*b* = 0.950, *p* < 0.01, 95% CI [0.328, 1.563]), Avoidant (*b* = 1.365, *p* < 0.01, 95% CI [0.492, 2.238]), and Dependent Personality (*b* = 1.153, *p* = 0.01, 95% CI [0.262, 2.043]). For models with interactions, higher levels of tau at baseline were significantly associated with increase in BPSD over time for all RSMB subdomains and Total RSMB (*b* = 1.305, *p* = 0.001, 95% CI [0.532, 2.078]). Figure 3 depicts the relation between baseline tau and average change in RSMB subdomains and Total RSMB across time, unadjusted for covariates. For visualization, participants are grouped by low versus high tau using SUVR threshold of 1.18 (i.e., average or above average/below average). Males had significantly lower Depression-Behavior (*b* = −0.308, *p* < 0.01, 95% CI [−0.511, −0.106]) and Dependent Personality (*b* = −0.356, *p* = 0.02, 95% CI [−0.653, −0.060]) scores than females. Those who were APOE ϵ4 allele carriers had higher Depression-Behavior (*b* = 0.253, *p* = 0.04, 95% CI [0.012, 0.493]) scores than non-carriers. Younger participants had significantly higher Restricted and Repetitive Behaviors (*b* = −0.016, *p* = 0.02, 95% CI [−0.028, −0.003]) scores than older participants, and these behaviors were reported more in participants with severe ID (*b* = 0.297, *p* = 0.04, 95% CI [0.014, 0.580]) than participants with mild ID. Psychiatric medication use was statistically significant in all models except for Depression-Behavior and Dependent Personality, and psychiatric diagnoses was significant in all models except for Aggression, Psychosis, and Paranoia.

**Table 4:** Multilevel models of tau at baseline predicting RSMB

	Estimates					
RSMB	Fixed Effects	*B*	*SE*	*t*	*df*	95% CI
Aggression	Intercept	−0.068	0.626	−0.110	136	[−1.307, 1.170]
	Time	−0.002	0.016	−0.150	327	[−0.035, 0.030]
	Tau	0.281	0.528	0.560	327	[−0.757, 1.319]
Restricted/Repetitive	Intercept	−0.594	0.323	−1.840	136	[−1.232, 0.044]
	Time	0.004	0.013	0.350	327	[−0.021, 0.030]
	Tau	0.540	0.271	1.990*	327	[0.006, 1.073]
Depression-Behavior	Intercept	−0.726	0.374	−1.940	136	[−1.466, 0.014]
	Time	0.018	0.019	0.97	327	[−0.019, 0.055]
	Tau	0.950	0.314	3.010**	327	[0.328, 1.563]
Depression-Physical	Intercept	−0.094	0.757	−0.120	136	[−1.591, 1.403]
	Time x Tau	0.515	0.158	3.270**	326	[0.205, 0.825]
Avoidant	Intercept	−1.280	0.528	−2.420*	136	[−2.324, −0.236]
	Time	0.047	0.021	2.210*	327	[0.005, 0.088]
	Tau	1.365	0.444	3.08**	327	[0.492, 2.238]
Dependent	Intercept	−0.855	0.538	−1.590	136	[−1.920, 0.209]
	Time	0.014	0.020	0.700	327	[−0.025, 0.054]
	Tau	1.153	0.453	2.550*	327	[0.262, 2.043]
Psychosis	Intercept	−0.392	0.445	−0.880	136	[−1.273, 0.489]
	Time x Tau	0.289	0.094	3.060**	326	[0.103, 0.475]
Paranoia	Intercept	−0.191	0.345	−0.550	136	[−0.874, 0.492]
	Time	0.023	0.014	1.620	327	[−0.005, 0.051]
	Tau	0.310	0.290	1.070	327	[−0.261, 0.881]
Total	Intercept	−1.470	2.224	−0.660	136	[−5.870, 2.925]
	Time x Tau	1.305	0.393	3.320**	326	[0.532, 2.078]

*N* = 143. Unstandardized coefficients (*B*s) are presented

All models include a random intercept and control for study site, age at baseline, sex, APOE ϵ4 carrier status, premorbid intellectual disability, psychiatric medications, and psychiatric diagnoses. Main effects were entered into models with interactions

*RSMB* Reiss Screen for Maladaptive Behavior

^***^ *p* < .001. ** *p* < .01. * *p* < .05

## Discussion

This longitudinal study investigated change in the frequency of reported RSMB scores to understand BPSD across AD progression in adults with DS by leveraging the ABC-DS study. Adults with DS who had MCI at baseline had higher RSMB scores than did adults with DS who were cognitively stable. The MCI group also showed greater increases in Total RSMB scores and specifically in Depression-Behavioral, Depression-Physical, and Psychosis RSMB scores across the 4 years than did the cognitively stable group. These findings highlight that changes in BPSD are a component of the prodromal stage of AD in DS, occurring prior to the onset of AD dementia. Thus, new or increased overreliance on others, restricted and repetitive behaviors, and social withdrawal present alongside early cognitive decline in adults with DS. Over time, changes in sadness and crying, eating and energy, and confused thinking may occur for adults with DS and MCI.

In models with main effects only, adults with DS who had AD dementia at baseline evidenced higher RSMB subdomains relative to adults with DS who were cognitively stable. AD dementia was not significant in any models with interactions between baseline clinical AD status and study cycle. That is, after AD dementia onset, on average, individual behaviors and symptoms appear to remain elevated over 48 months and do not increase more. However, prior work [41, 43] indicates that this may be the case for overall BPSD, but not specific BPSD (e.g., some become elevated earlier while others become elevated later). These findings indicate a critical need for care planning and management of these symptoms whenever they appear.

Elevated Aβ and NFT were associated with higher baseline Avoidant RSMB scores, and an increase in Depression-Physical RSMB scores over time. These findings expand on previous research that avoidant behaviors (e.g., social withdrawal) and depression (e.g., low energy, lethargic) present early on in AD progression in DS [64–67], and it is possible that Aβ and NFT are neurobiological mechanisms that drive these behaviors, as has been theorized [28, 68]. These BPSD have also been reported to be associated with elevated Aβ [29] and NFT [28] in the general adult population [23].

Across models, sociodemographic characteristics were related to RSMB scores. Females with DS exhibited higher behavioral symptoms of depression (e.g., tearfulness, overt sensitivity) than males. This sex difference has been previously reported in adults with DS [69] and in the general adult population [70] and has important implications for BPSD screening. APOE ϵ4 allele carriers had lower aggression symptoms over time in clinical AD status and Aβ models. There was a negative association between age and restricted and repetitive behaviors in Aβ and NFT models.

The present study had both strengths and limitations. The study is the first, to our knowledge, longitudinally investigate of the relation between neuroimaging biomarkers of early AD pathology, clinical AD status, and changes in BPSD in adults with DS. Study strengths also include the use of a large cohort of adults with DS from whom clinical AD status was based on a robust battery of direct and informant measures and made in consideration of medical history, premorbid ID level, and recent life events. The RSMB was designed to measure maladaptive behaviors in individuals with ID and to distinguish symptoms from cognitive impairments, but was not specifically developed to screen for BPSD with AD. In addition, the RSMB assesses a limited number of symptoms and captures a restricted severity range (i.e., symptoms are rated on a three-point Likert scale) and thus may not have been sensitive to subtle changes in BPSD over the four data collection cycles. The broader array of BPSD reported in prior studies on AD in DS were not captured by the RSMB, nor was the RSMB able to provide in depth information on type of symptoms (e.g., apathy versus other behavioral depressive symptoms) [37, 43]. The RSMB is also open to potential biases as it is an informant report of internalizing and externalizing behaviors. Future studies should draw on self-report and structured clinical interviews of BPSD. The present study did not account for potential changes in specific psychiatric medications (e.g., antipsychotics, antidepressants) across study cycles, which could have reduced our ability to detect symptom increases. It should also be noted that scores on the RSMB were made available during the clinical AD status consensus meetings, introducing potential confounding. However, clinical AD status decisions were based on gestalt impressions drawing on multiple cognitive and informant measures.

Additionally, there was a lack of racial and ethnic diversity in the sample, and participants with mild ID and/or were younger were overrepresented in the ABC-DS sample. The age difference between participants with versus without PET scans is likely a reflection of study design; the ABC-DS study merges two legacy studies—the study that conducts the most brain imaging historically had a lower age range for study inclusion. Study sites have efforts underway to increase sample diversity as this is critical for the field moving forward. Further research should also examine whether Aβ or NFT burden in specific brain regions drives BPSD as AD unfolds in DS.

In conclusion, findings from the present study have important implications for screening and diagnosis of MCI and AD dementia in adults with DS. Findings also have relevance for directing interventions for care planning and alleviating BPSD at different stages in AD progression in adults with DS. Increases in social withdrawal, low energy, and lethargy may indicate that an adult with DS is in the preclinical stage of AD (evident by elevated Aβ and tau). New or increased difficulties in these behaviors may appear at the time of MCI with subsequent increases more broadly in BPSD occurring from early to late stages of dementia. Screening protocols for AD in adults with DS should differentiate psychiatric causes of dementia-like symptoms, ideally with measures developed for assessing BPSD. However, it is also important for clinicians to rule out other causes for BPSD, as new or increases in symptoms can also occur outside of AD pathology and be due to factors such as life experiences (recent stressful life events) or other co-occurring medical or biological conditions (e.g., thyroid conditions, pain). It is also important for caregivers of adults with DS to be educated about the types of BPSD that are a common part of AD and strategies for managing these symptoms, including not only pharmaceutical options but also behavior management techniques, safety planning, and self-care for the caregiver.

## Supplementary Information

Supplementary Material 1

## Data Availability

The data analyzed in this study was obtained from The National Institutes of Health (NIH) National Institute on Aging (NIA) Alzheimer’s Biomarkers Consortium—Down Syndrome (ABC-DS), the following licenses/restrictions apply: Applicants must complete and submit a data request form (ABC-DS Data Request Form) and review and sign the ABC-DS Data Use Agreement. Requests to access these datasets should be directed to ABC-DS, https://www.nia.nih.gov/research/abc-ds.

## Abbreviations

DS: Down syndrome
AD: Alzheimer’s disease
MCI: Mild cognitive impairment
Aβ: Amyloid-beta
NFT: Neurofibrillary tangles of tau
