# Supplementary material for: Behavioral and psychological symptoms of dementia and Alzheimer’s disease progression in Down syndrome
Source: J Neurodev Disord. 2025 Apr 11;17:19. doi: 10.1186/s11689-025-09604-w (PMC11987311; doi:10.1186/s11689-025-09604-w)
Supplement: Supplementary file 1 — Supplementary Material 1 [file 11689_2025_9604_MOESM1_ESM.docx]

Supplemental Statistical Models

Models included a fixed effect of time, age at baseline, biological sex, APOE4 status, premorbid ID, psychiatric medication use, and psychiatric diagnosis, as well as a random intercept to account for within-subject correlation. A fixed interaction effect between time and baseline clinical AD status was used to explore the differences in BPSD trajectories between cognitively stable participants and the groups a) MCI, and b) dementia. Individuals were nested within study sites. The following statistical model was fit for each BPSD (y):

$$y_{tij}= \beta_{0}+ \beta_{1}{Time}_{tij}+\beta_{2}{Clinical AD Status}_{i}+\beta_{3}{Clinical AD Status}_{i}+ \beta_{4}{Clinical AD Status}_{i} \times{Time}_{tij}+ \beta_{5}{Clinical AD Status}_{i} \times{Time}_{tij}+\beta_{6}{Sex}_{i}+\beta_{7}{Age}_{i}+\beta_{8}{Premorbid ID}_{i}+\beta_{9}{Premorbid ID}_{i}+\beta_{10}{APOE4}_{i}+\beta_{11}{Medication}_{ti}+\beta_{12}{Diagnosis}_{ti}+ u_{j}+ r_{0ij}+e_{tij}$$

where:

1. $\beta_{0}$ is the intercept (average BPSD for reference group [cognitively stable] at baseline);
2. ${Time}_{tij}$ is the effect of time in years, fitted as continuous;
3. ${Clinical AD Status}_{i}$ is for participant ${Clinical AD Status}_{i}$ = 0 if status is cognitively stable, 1 if status is MCI, and 2 if status is dementia;
4. ${Sex}_{i}$ is for biological sex for participant ${Sex}_{i}$ = 0 if female, 1 if male;
5. ${Age}_{i}$ is age at baseline for subject *i ;*
6. ${Premorbid ID}_{i}$ is premorbid ID for participant ${Premorbid ID}_{i}$ = 0 if mild ID, 1 if moderate ID, and 2 if severe ID;
7. ${APOE4}_{i}$ is APOE4 status for participant ${APOE4}_{i}$ = 0 if not carrier, 1 if carrier;
8. ${Medication}_{ti}$ is any psychiatric medication use at time *t* for participant ${Medication}_{i}$ = 1 if yes, 0 if no;
9. ${Diagnosis}_{ti}$ is any psychiatric diagnosis at time *t* for participant ${Diagnosis}_{i}$ = 1 if yes, 0 if no;
10. $u_{j}$ is the group effect accounting for stable mean-level differences in *y* across study sites;
11. $r_{0ij}$ is the random intercept accounting for participant differences in *y* at baseline (around $\beta_{0}+ u_{j}$);
12. $e_{tij}$ is the residual accounting for time-specific variation in *y* around participant’s growth trajectories

Random effects and residuals are assumed to be independent and normally distributed, such that

$$u_{j} \sim N\left( 0,\sigma_{u}^{2} ) r_{0ij}\sim N(0, \sigma_{r0}^{2} \right) e_{tij} \sim N(0,\sigma_{e}^{2})$$

Interaction terms between time and each AD biomarker were used to explore the differences in BPSD with respect to Aβ and NFT. As with the prior model, individuals were nested within study site. In the following statistical model, parameters are defined the same as above except for $\beta_{2}$ through $\beta_{5}$:

$$y_{tij}= \beta_{0}+ \beta_{1}{Time}_{tij}+\beta_{2}{Amyloid Beta}_{i}+ \beta_{3}{Amyloid Beta}_{i} \times{Time}_{tij}+\beta_{4}{Sex}_{i}+\beta_{5}{Age}_{i}+\beta_{6}{Premorbid ID}_{i}+\beta_{7}{Premorbid ID}_{i}+\beta_{8}{APOE4}_{i}+\beta_{9}{Medication}_{ti}+\beta_{10}{Diagnosis}_{ti}+ u_{j}+ r_{0ij}+e_{tij}$$

where

1. ${Amyloid Beta}_{i}$ is the baseline Aβ for participant *i*, expressed as centiloids and fitted as continuous

$$y_{tij}= \beta_{0}+ \beta_{1}{Time}_{tij}+\beta_{2}{NFT}_{i}+ \beta_{3}{NFT}_{i} \times{Time}_{tij}+\beta_{4}{Sex}_{i}+\beta_{5}{Age}_{i}+\beta_{6}{Premorbid ID}_{i}+\beta_{7}{Premorbid ID}_{i}+\beta_{8}{APOE4}_{i}+\beta_{9}{Medication}_{ti}+\beta_{10}{Diagnosis}_{ti}+ u_{j}+ r_{0ij}+e_{tij}$$

where

1. ${NFT}_{i}$ is the baseline neurofibrillary tau tangles (NFT) for participant *i*, expressed as standard uptake value ratios (SUVR) in Braak stages I and II and fitted as continuous

Supplemental Table 1

| Average BPSD Across Time by Baseline Clinical AD Status | | | | |
| --- | --- | --- | --- | --- |
|  | 0 Months | 16 Months | 32 Months | 48 Months |
|  | M (SD) | M (SD) | M (SD) | M (SD) |
| RSMB | Cognitively Stable | | | |
| Aggression | 0.43 (1.11) | 0.30 (0.97) | 0.35 (0.97) | 0.30 (0.87) |
| Restricted/Repetitive | 0.25 (0.69) | 0.28 (0.71) | 0.30 (0.70) | 0.30 (0.81) |
| Depression-Behavior | 0.46 (1.08) | 0.43 (0.84) | 0.42 (0.87) | 0.37 (1.05) |
| Depression-Physical | 0.74 (1.32) | 0.65 (1.07) | 0.66 (1.20) | 0.68 (1.18) |
| Avoidant | 0.41 (0.82) | 0.46 (1.06) | 0.49 (0.90) | 0.56 (1.13) |
| Dependent | 0.59 (1.15) | 0.55 (1.03) | 0.54 (1.16) | 0.43 (0.82) |
| Psychosis | 0.32 (0.83) | 0.24 (0.59) | 0.30 (0.79) | 0.37 (0.96) |
| Paranoia | 0.32 (0.79) | 0.27 (0.68) | 0.29 (0.68) | 0.30 (0.88) |
| Total | 2.47 (4.03) | 2.18 (3.33) | 2.32 (3.47) | 2.26 (3.94) |
|  | MCI | | | |
| Aggression | 0.69 (1.43) | 0.73 (1.51) | 0.64 (1.30) | 0.93 (1.64) |
| Restricted/Repetitive | 0.56 (0.92) | 0.52 (0.90) | 0.67 (1.20 | 0.64 (0.93) |
| Depression-Behavior | 0.71 (1.41) | 0.67 (1.10) | 1.10 (1.59) | 1.36 (2.27) |
| Depression-Physical | 1.15 (1.35) | 1.27 (1.55) | 1.62 (1.77) | 1.79 (2.15) |
| Avoidant | 0.69 (1.22) | 1.02 (1.51) | 1.24 (1.57) | 1.00 (1.62) |
| Dependent | 0.85 (1.40) | 0.83 (1.56) | 0.76 (1.05) | 0.79 (0.97) |
| Psychosis | 0.54 (0.90) | 0.75 (1.26) | 0.93 (1.39) | 1.21 (1.53) |
| Paranoia | 0.58 (1.24) | 0.60 (1.14) | 0.64 (1.06) | 0.71 (1.90) |
| Total | 3.90 (4.80) | 4.50 (5.35) | 5.45 (5.18) | 6.21 (7.92) |
|  | Dementia | | | |
| Aggression | 1.33 (2.18) | 1.23 (2.14) | 0.73 (1.32) | 1.00 (1.41) |
| Restricted/Repetitive | 0.48 (0.77) | 0.48 (0.78) | 0.32 (0.89) | 0.57 (0.79) |
| Depression-Behavior | 1.02 (1.09) | 0.95 (1.30) | 0.86 (1.39) | 0.14 (0.38) |
| Depression-Physical | 1.76 (2.09) | 2.20 (2.32) | 1.18 (1.92) | 1.71 (1.80) |
| Avoidant | 0.98 (1.26) | 1.23 (1.80) | 0.82 (1.68) | 0.71 (0.95) |
| Dependent | 1.05 (1.29) | 1.35 (1.81) | 1.09 (1.48) | 1.00 (1.29) |
| Psychosis | 1.02 (1.07) | 1.38 (1.63) | 1.00 (1.51) | 0.86 (1.21) |
| Paranoia | 0.62 (1.19) | 0.85 (1.35) | 0.68 (1.21) | 0.57 (1.13) |
| Total | 6.31 (5.97) | 7.23 (7.53) | 5.09 (6.78) | 5.14 (5.15) |
| Note. BPSD = Behavioral and Psychological Symptoms of Dementia. RSMB = Reiss Screen for Maladaptive Behavior. AD = Alzheimer’s Disease. MCI = Mild Cognitive Impairment. | | | | |

Supplemental Table 2

| Frequencies of Clinical AD Status Across Time by Baseline Clinical AD Status | | | | | |
| --- | --- | --- | --- | --- | --- |
|  |  | Baseline Clinical AD Status | | | |
|  |  | Cognitively stable | MCI | Dementia | Total |
| 16 months | Cognitively stable | 222 | 4 | 0 | 226 |
|  | MCI | 17 | 19 | 0 | 36 |
|  | Dementia | 0 | 24 | 40 | 64 |
|  | Unable to determine | 8 | 1 | 2 | 11 |
|  | Total | 247 | 48 | 42 | 337 |
| 32 months | Cognitively stable | 180 | 2 | 1 | 183 |
|  | MCI | 18 | 9 | 0 | 27 |
|  | Dementia | 9 | 30 | 20 | 59 |
|  | Unable to determine | 6 | 1 | 1 | 8 |
|  | Total | 213 | 42 | 22 | 277 |
| 48 months | Cognitively stable | 93 | 3 | 1 | 97 |
|  | MCI | 12 | 1 | 0 | 13 |
|  | Dementia | 8 | 9 | 6 | 23 |
|  | Unable to determine | 2 | 1 | 0 | 3 |
|  | Total | 115 | 14 | 7 | 136 |
| Note. AD = Alzheimer’s Disease. MCI = Mild Cognitive Impairment. | | | | | |
